# Induction of apoptosis in cancer cell lines by the Red Sea brine pool bacterial extracts

**DOI:** 10.1186/1472-6882-13-344

**Published:** 2013-12-05

**Authors:** Sunil Sagar, Luke Esau, Karie Holtermann, Tyas Hikmawan, Guishan Zhang, Ulrich Stingl, Vladimir B Bajic, Mandeep Kaur

**Affiliations:** 1 Computational Bioscience Research Center, King Abdullah University of Science and Technology (KAUST), Thuwal 23955-6900, Kingdom of Saudi Arabia; 2 Red Sea Research Center, King Abdullah University of Science and Technology (KAUST), Thuwal 23955-6900, Kingdom of Saudi Arabia

**Keywords:** Natural products, Cancer, Apoptosis, Marine bacteria, Deep-sea brine pools

## Abstract

**Background:**

Marine microorganisms are considered to be an important source of bioactive molecules against various diseases and have great potential to increase the number of lead molecules in clinical trials. Progress in novel microbial culturing techniques as well as greater accessibility to unique oceanic habitats has placed the marine environment as a new frontier in the field of natural product drug discovery.

**Methods:**

A total of 24 microbial extracts from deep-sea brine pools in the Red Sea have been evaluated for their anticancer potential against three human cancer cell lines. Downstream analysis of these six most potent extracts was done using various biological assays, such as Caspase-3/7 activity, mitochondrial membrane potential (MMP), PARP-1 cleavage and expression of γH2Ax, Caspase-8 and -9 using western blotting.

**Results:**

In general, most of the microbial extracts were found to be cytotoxic against one or more cancer cell lines with cell line specific activities. Out of the 13 most active microbial extracts, six extracts were able to induce significantly higher apoptosis (>70%) in cancer cells. Mechanism level studies revealed that extracts from *Chromohalobacter salexigens* (P3-86A and P3-86B(2)) followed the sequence of events of apoptotic pathway involving MMP disruption, caspase-3/7 activity, caspase-8 cleavage, PARP-1 cleavage and Phosphatidylserine (PS) exposure, whereas another *Chromohalobacter salexigens* extract (K30) induced caspase-9 mediated apoptosis. The extracts from *Halomonas meridiana* (P3-37B), *Chromohalobacter israelensis* (K18) and *Idiomarina loihiensis* (P3-37C) were unable to induce any change in MMP in HeLa cancer cells, and thus suggested mitochondria-independent apoptosis induction. However, further detection of a PARP-1 cleavage product, and the observed changes in caspase-8 and -9 suggested the involvement of caspase-mediated apoptotic pathways.

**Conclusion:**

Altogether, the study offers novel findings regarding the anticancer potential of several halophilic bacterial species inhabiting the Red Sea (at the depth of 1500–2500 m), which constitute valuable candidates for further isolation and characterization of bioactive molecules.

## Background

Over the last four decades, natural products have played an important role in drug discovery against cancer, one of the deadliest diseases in the world and the second most common cause of death in developed countries. Almost 47% of the anticancer drugs approved in the last 50 years were either natural products or synthetic molecules inspired by natural products [1]. However, due to high toxicity and undesirable side effects associated with cancer drugs and, in particular, due to the development of resistance to chemotherapeutic drugs, there is a continuous need for novel drugs with greater therapeutic efficiency and/or with fewer side effects [2].

Marine microorganisms are considered to be an important source of bioactive molecules against various diseases and have great potential to increase the number of lead molecules in clinical trials. Approximately 3000 natural products have been isolated from marine microbial/algal sources and are described in Antibase [3]. Several of these microbial natural products have been evaluated in clinical trials for the treatment of various cancers [4,5]. Two cyanobacteria-derived antimicrotubule agents, i.e. dolastatin A and curacin A have been clinically evaluated against cancer and served as a lead structure for the synthesis of number of synthetic analogs/derivatives [6]. Another compound, salinosporamide A, isolated from a marine-derived actinomycete, a highly potent irreversible inhibitor of 20S proteasome, was also used in clinical trials as an anticancer agent [7]. Additionally, there is circumstantial evidence that several lead molecules in the clinical development pipeline, thought to originate from higher marine organisms, may actually be produced by marine microbes [8].

In the last decade, the deep sea has emerged as a new frontier in the isolation and screening of natural products, especially for cancer research [9,10]. With advancements in technology leading to greater accessibility as well as improvements in techniques used to culture microorganisms, deep sea environments are becoming ‘hot spots’ for new and unexplored chemical diversity for drug discovery [11]. Approximately 30,000 natural products have been isolated from marine organisms, yet less than 2% of those derive from deep-water marine organisms (http://dmnp.chemnetbase.com/intro/index.jsp). Of these, several cytotoxic secondary metabolites isolated from deep sea microorganisms have been described in the literature [9,12-14]. In our own efforts in this direction, cytotoxic and apoptotic potentials of marine halophilic bacteria isolated from the deep sea brine pools of the Red Sea, a largely unexplored resource in the area of natural product research, have been described previously [15], including the first report of cytotoxic activity of ‘*Sulfitobacter’*.

In the present study, ethyl acetate extracts of 24 marine bacterial strains, isolated from the deep sea brine pools of the Red Sea, have been evaluated for their anticancer potential against HeLa (cervical carcinoma), DU145 (prostate carcinoma), and MCF-7 (breast adenocarcinoma) cell lines. The rationale behind choosing the cell lines lie in the severity and/or prevalence of various cancers in Saudi Arabia as well as around the world. The prevalence of breast cancer in the Kingdom of Saudi Arabia has increased from 10.2% in 2000 to 47.2% in 2007 [16]. Similarly, a screening program has demonstrated higher prevalence of prostate cancer [17] in the Kingdom. Another study anticipated a significant increase in proportion of cervical cancer [18] cases in the Kingdom. These cells lines are the also among the most robust cell line models used for *in vitro* drug screening. The evaluation of proapoptotic potential of highly cytotoxic extracts further revealed six highly potent extracts that were subjected to more detailed assays to infer the pathways involved in apoptotic mode of cell death in cancer cells.

## Methods

### Field sampling

The samples were retrieved from brine-seawater interfaces, brine layers, and sediments of deep-sea brine pools during KAUST Red Sea Expedition 2011. Water samples were collected using a rosette sampler equipped with 20 Niskin bottles (10 liter each) and a conductivity-temperature-depth (CTD) unit for monitoring salinity, temperature, transmission, and pressure (Idronout S.r.l, Italy). At each sampling site, approximately 180 litres of sample were collected and pre-filtered through a 5.0 μm SMWP membrane (diameter 290 mm; Millipore, Ireland) to remove suspended particles. A tangential flow filtration (TFF) system (Pellicon 2 Filter Acrylic Holder, Millipore, US) was used in order to filter-concentrate the samples. One liter of each concentrated sample was obtained after retention through a Durapore 0.1 μm PVDF filter (Pellicon 2 Cassette Filter, Screen type C, size 0.5 m2, Millipore Corporation, MA, USA). These concentrated samples were stored in a dark bottle at 4°C, and used as inoculum for microbial isolations. Sediment collection was performed by deploying a multicore sampling device into the bottom of the brine pools. The top layer of sediment (approximately 10 cm) was cut, kept in anoxic containers in the dark, and were later used as inoculum for microbial isolations.

### Source of bacterial isolates

A total of 24 bacterial strains were successfully isolated from deep-sea brine pools of the Red Sea. Nineteen of them were isolated from brine-seawater interfaces, one strain from brine, and four strains from sediments (Table 1). The inocula for bacterial isolation were collected from five different brine pools named Atlantis II (2194 m), Discovery Deep (2224 m), Kebrit Deep (1573 m), Nereus Deep (2458 m), and Erba Deep (2395 m) [19]. Each of the deep-sea brine pools has its unique physicochemical composition, with salinity up to 26%, including notably high temperature, as well as high concentrations of heavy metals [20]. The Atlantis II Deep and the Discovery deep are considered as hot brines, with maximum temperatures 67.8°C and 44.8°C, respectively. Brine–seawater boundaries in these brine pools are characterized by strong temperature and salinity gradients [21].

**Table 1:** Taxonomic identification and collection location for 24 microbial strains

**Isolates**	**Source**	**Closest relative**	**Similarity (16S rRNA genes)**	**Accession number**	**Ref.**
P1-16A	Kebrit interface	*Halomonas meridiana*	100%	AF212217	[22]
P1-16C	Kebrit interface	*Idiomarina loihiensis*	98%	AE017340	[23]
P1-26A	Nereus brine	*Virgibacillus salarius*	98%	NR_041270	[24]
P1-5	Kebrit interface	*Sediminimonas qiaohouensis*	99%	NR_044577	[25]
P2-13A	Kebrit sediment	*Chromohalobacter salexigens*	99%	CP000285	[26]
P2-13B	Kebrit sediment	*Halobacillus halophilus*	99%	HE717023	[27]
P2-16A	Kebrit interface	*Halomonas meridiana*	99%	AF212217	[22]
P3-16A	Kebrit interface	*Halomonas meridiana*	99%	AF212217	[22]
P3-16B	Kebrit interface	*Halomonas meridiana*	99%	AF212217	[22]
P3-37A	Nereus interface	*Halomonas meridiana*	99%	AF212217	[22]
P3-37B	Nereus interface	*Halomonas meridiana*	99%	AF212217	[22]
P3-37C	Nereus interface	*Idiomarina loihiensis*	91%	AE017340	[23]
P3-86A	Discovery interface	*Chromohalobacter salexigens*	100%	CP000285	[26]
P4-13A	Kebrit sediment	*Chromohalobacter salexigens*	99%	CP000285	[26]
P4-13B	Kebrit sediment	*Staphylococcus* sp.	100%	JQ082193	[28]
P5-86A	Discovery interface	*Idiomarina baltica*	99%	NR_027560	[29]
P5-86B	Discovery interface	*Chromohalobacter salexigens*	99%	CP000285	[26]
P6-86	Discovery interface	*Chromohalobacter salexigens*	99%	CP000285	[26]
K-2	Atlantis II interface	*Zunongwangia profunda*	100%	NR_074656	[30]
K-18	Atlantis II interface	*Chromohalobacter israelensis*	100%	AM945672	[31]
K-30	Atlantis II interface	*Chromohalobacter salexigens*	99%	CP000285	[26]
H102	Erba brine interface	*Marinobacter adhaerens*	100%	NR_074765	[32]
H105	Erba brine interface	*Idiomarina zobellii*	99%	NR_024892	[33]
P3-86B (2)	Discovery interface	*Chromohalobacter salexigens*	96%	CP000285	[26]

All of the bacterial strains isolated in this study were obtained by the streak plate method described elsewhere [34]. Eighteen strains grew in salinities of 10% NaCl and the rest of the isolates grew well in salinities of 20% NaCl.

### PCR amplification

Nucleic acids were extracted with Qiagen kit (DNeasy blood & tissue kit, Qiagen, Germany) according to the instruction manual. PCR amplifications of the extracted DNA were performed in a 25 μl reaction, each mixture containing 12.5 μl Promega PCR Master Mix 2x (Promega, USA), 1 μl (final concentration 0.5 μM) of primer 27bF (5′-AGAGTTTGATCMTGGCTCAG-3′) and 1492uR (5′-TACCTTGTTACGACTT-3′), 8.5 μl RNAase&DNAase free H2O (Teknova), and DNA template. PCR was carried out in Mastercycler (Eppendorf, Germany) under following conditions: 94°C for 3 min; 35 cycles of 94°C for 60 s, 53°C for 90 s, 72°C for 90 s. A final extension was done for 7 min at 72°C. The yield and quality of the PCR products were examined on 1% (wt/vol) agarose gel (SeaKem GTG, Lonza, USA) stained with SYBR Safe (Invitrogen, USA). All sequencing reactions were purified with Illustra Exostar 1-step (GE, Healthcare, UK) according to the manufacturer’s protocol. The 16S rRNA sequences were determined using an ABI 3730xl capillary DNA sequencer (PE Applied Biosystems), at Core Laboratory KAUST, Saudi Arabia.

### Bacterial biomass

The concentrated samples were inoculated onto three different agar media, plate count agar (Teknova), marine agar 2216 (Difco), and R2A agar (Oxoid), which were supplemented with either 10% or 20% NaCl (w/v) to adjust salinity. The plates were incubated at 30°C for up to three weeks and inspected daily. Colonies from various agar plates were picked based on difference in colony morphology. Pure isolates of these colonies were obtained after three successive transfers to the fresh agar media. Taxonomic identifications of the isolates were based on 16S rRNA gene sequencing. 16S rRNA gene amplification and sequencing steps were performed according to [35]. Sequence similarity was analyzed using BLASTN search program to identify the strains to their closest relatives in GenBank database (http://www.ncbi.nlm.nih.gov/BLAST). Bacteria were inoculated in 1 liter of Marine Broth (Difco) supplemented with NaCl to collect the biomass, and then were incubated at 30°C in a shaking incubator. After two weeks of incubation, bacterial cultures were harvested by centrifugation at ambient temperature for an hour (h). The centrifugation step was repeated by adding sterile water at the same salinity to wash the pellets. Cell pellets were stored at −80°C until used for extract preparation.

### Extract preparation

Ethyl acetate extracts of 24 strains of marine bacteria were prepared at a concentration of 100 mg/mL (speedvac dried material/mL of solvent). Solutions were sonicated with ultra-sound probe (Biologics Inc., Model 150 V/T) for 5 × 2 minutes on ice. The solutions were centrifuged at 10000 g for 15 minutes; the supernatants were recovered and stored at −20°C.

### Cell culture

MCF-7 (Breast Adenocarcinoma), HeLa (Cervical carcinoma), and DU145 (Prostate carcinoma) were obtained from the American Type Cell Culture Collection (ATCC, Manassas, VA). All cell lines were cultured in DMEM (Dulbecco’s Modified Eagle’s Medium), supplemented with 10% FCS (Fetal calf serum), penicillin (100 U/mL) and streptomycin (100 μg/mL) at 5% CO_2_ in a 37°C incubator.

### MTT assay

The cytotoxicity of marine bacterial extracts was estimated by MTT (3-(4, 5-Dimethylthiazol-2-yl)-2, 5-diphenyltetrazolium bromide) assay. Cells were seeded at a density of 2.5 × 10^3^ cells per well in a 384-well culture plates and treated with 200 and 500 μg/mL marine bacterial extracts for 48 h. Following incubation with extracts, 5 μL of sterile MTT (5 mg/mL) dissolved in PBS was added to each well and incubated with cells for 4 h followed by the addition of 30 μL of solubilization solution (10% SDS, 10 mM HCl), which was further incubated with cells for 16 h at 37°C. The OD (optical density) of each well was measured at 595 nm using a microtiter plate reader (BMG Labtech PHERAstar FS, Germany) and results were analyzed using Microsoft Office Excel©.

### APOPercentage assay

HeLa cells were seeded in 96-well plates at a density of 5 × 10^3^ cells per well in quadruplicate in 90 μL of media. After 24 h, cells were treated with marine bacterial extracts diluted in complete DMEM to a final concentration of 500 μg/mL and incubated at 37°C for 24 and 48 h. Cells were treated with 10 mM H_2_O_2_ for 30 minutes as a positive control. The cells were lifted and stained with APOPercentage dye (Biocolor, UK). Percentage of cells stained positive for apoptosis was determined with a high throughput flow cytometer (HTFC) Screening System (IntelliCyt Corporation, Albuquerque, NM). Cells were gated for FSC-H, SSC-H and in the FL-2H channel recording a minimum of 1000 events per well.

### Microscopy

The morphological evaluation and photography of cells after treatment with extracts was done in 96-well plates using Primo Vert inverted microscope (Carl Zeiss, Inc.)

### MMP assay

HeLa cells were seeded in 96-well plates at a density of 5 × 10^3^ cells per well in quadruplicate in 90 μL of media and allowed to settle overnight. Next day, cells were treated with 500 μg/mL marine bacterial extracts for 12 and 16 h and stained with 50 μM cyanine dye JC-1 (5,5′,6,6′-tetrachloro-1,1′,3,3′-tetraethylbenzimi- dazolylcarbocyanine iodide, Life Technologies, UK) for 1 h. Cells were analyzed by HTFC system by plotting FL2-H vs. FL-1H and applying a quadrant gate to determine JC-1 aggregates (red) and monomers (green).

### Caspase assay

HeLa cells were seeded at a density of 2.5 × 10^3^ cells per well in 20 μL of media in 384-well plates. After 24 h, 5 μL of marine bacterial extract (500 μg/mL) was added and incubated for a further 16 h. Caspase-3/7 activity was estimated using ApoTox-Glo kit (Promega) following the manufacturer’s instructions. Luminescence was measured using a luminescence plate reader (BMG Labtech PHERAstar FS, Germany). The results were normalized to cell viability (measured using MTT assay).

### Western blotting

HeLa cells were seeded at a density of 3 × 10^5^ cells per well in 6-well plates and left overnight to settle. Cells were treated with 500 μg/mL of marine bacterial extracts for 12 and 24 h. Protein was harvested with RIPA lysis buffer (150 mM NaCl, 1% Triton X 100, 0.1% SDS, 10 mM Tris pH 7.5, 1% sodium deoxycholate) and quantitated with a BCA protein determination kit (Pierce Thermo Scientific). 10–20 μg of protein lysate was subjected to electrophoresis on 12% SDS page gels, transferred to nitrocellulose membrane and probed with Caspase-8 (Sigma), Caspase-9 (Thermo Scientific), PARP-1 (Trevigen) and pH2Aγ (Enzo Life Technologies) antibodies. β-Tubulin (Santa Cruz) was used as a loading control.

### Z-factor

Z-factor was determined for each assay and a Z-factor score of ≥0.6 was recorded indicating good to excellent robustness for assays [36].

## Results

### Microbial isolates from the Red Sea

Twenty-four strains of marine bacteria were isolated from the samples collected from brine-seawater interfaces, brine layers, and sediments of five deep-sea brine pools of the Red Sea. Taxonomic classification and location of collection for these microbial strains is presented in Table 1. The samples were extracted by using ethyl acetate and evaluated for their anticancer potential through various biological assays.

### Antiproliferative activities of marine bacterial extracts

The antiproliferative effect of 24 marine bacterial extracts was evaluated *in vitro* by MTT assay against three human cancer cell lines, i.e. DU145 (prostate cancer), MCF-7 (breast cancer) and HeLa (cervical cancer). The cancer cells were exposed to marine extracts for 48 h (Table 2), at the concentrations of 200 and 500 μg/mL. In general, most of the microbial extracts were able to induce growth inhibition in one or more cancer cell line/s; however, extracts P1-5, P2-13B, P3-37B, H-102, P3-86B and P3-86A displayed up to 60% growth inhibition in DU145 cell line at 500 μg/mL. Similarly in MCF-7 cells, several microbial extracts were found to be cytotoxic at the same concentration. HeLa emerged as the most sensitive cell line as 13 microbial extracts inhibited 30% or more cell growth at 500 μg/mL concentration (Table 2). Extracts from *Halomonas meridiana* (P3-37B) and *Chromohalobacter salexigens* (K-30 and P3-86B(2)) displayed the highest growth inhibition, i.e. > 85%. Microbial extracts with more than 30% growth inhibition were chosen for further apoptotic analysis. HeLa was chosen for the downstream analysis of selected microbial extracts due to its higher sensitivity to most of the extracts.

**Table 2:** Evaluation of antiproliferative effects of marine bacterial extracts on various cancer cell lines

**Strain**	**HeLa**		**MCF-7**		**DU145**	
	**200 μg/mL**	**500 μg/mL**	**200 μg/mL**	**500 μg/mL**	**200 μg/mL**	**500 μg/mL**
P1-16A	8.0	16.2	0	0	3.2	1.0
**P3-16A**	12.9	32.8	0	0	4.9	6.1
P5-86B	2.8	2.8	0	0	0.8	8.3
**H102**	54.6	37.0	21.7	18.0	3.0	2.0
P3-16B	0	0	0	0	11.7	14.4
P6-86	0	0	0	0	2.0	6.8
P2-16A	0	0	0	0	7.3	6.0
P1-26A	0	0	0	0	3.9	2.7
P5-86A	0	0	0	0	1.4	10.2
**P3-86B(2)**	0	95.2	0	0	4.5	7.2
**P3-86A**	31.9	39.8	32.1	35.3	2.3	3.0
**P1-5**	27.2	56.3	33.4	54.0	52.1	59.9
**P1-16C**	30.7	35.3	33.5	51.1	41.4	47.0
H105	17.1	19.0	16.3	23.4	7.4	7.5
**P3-37A**	1.1	13.6	16.4	28.5	24.3	28.8
K2	1.4	15.5	13.2	12.4	14.6	11.9
P4-13A	0	6.9	4.2	3.8	0	12.3
**P3-37C**	0	4.9	22.1	34.8	21.7	1.5
**K18**	16.0	36.6	11.6	37.0	24.1	45.3
**P3-37B**	12.3	43.6	32.5	89.8	41.8	34.6
**K30**	24.7	28.4	27.9	88.2	18.6	30.4
**P2-13B**	35.3	50.5	39.5	51.4	62.5	60.7
**P4-13B**	8.2	8.4	29.0	37.7	44.7	39.8

The growth of cells was inhibited in a dose-dependent manner at concentrations of 200 and 500 μg/mL after 48 h. MTT assay was used to assess the growth inhibitory potential of extracts and is presented as percent growth inhibition relative to untreated cells. The Z-factor for this experiment was calculated to be 0.76. The extracts selected for apoptosis analysis are presented in bold letters.

### Apoptotic cell death in HeLa cells

**Figure 1:**
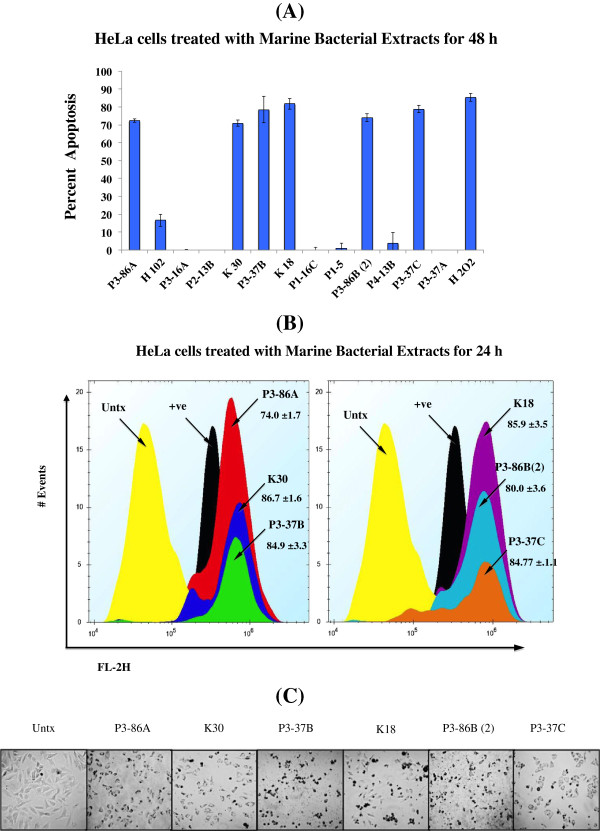
**Apoptosis induction in HeLa cells after treatment with marine bacterial extracts.** Detection of externalization of PS from cell membrane was done using APOPercentage assay. Cells were stained with dye and fluorescence was measured using flow cytometry. The photographs were taken by using inverted microscope. Apoptosis is shown as the percentage of cells that uptake APOPercentage dye relative to untreated cells. **A)** Cells were treated with H_2_O_2_ or extracts at a final concentration of 500 μg/mL for 48 h, and **B)** for 24 h. The Z- factor was calculated to be 0.68 and 0.77 for 48 h and 24 h experiments, respectively. **C)** The morphology of cancer cells after 24 h of treatment with chosen extracts.

Since anticancer agents are known to induce apoptosis in cancer cells and apoptosis biomarkers are being increasingly used in clinical trials [37], a total of 13 extracts showing significant cytotoxicity were tested for their proapoptotic potential in HeLa cells by using APOPercentage assay. Seven extracts were found to induce apoptosis at 500 μg/mL concentration after 48 h (Figure 1A). Extracts from *Chromohalobacter salexigens* (P3-86A, K30, P3-86B(2)), *Chromohalobacter israelensis* (K18), *Halomonas meridiana* (P3-37B) and *Idiomarina loihiensis* (P3-37C) induced more than 70% apoptosis in HeLa cells. Therefore, six most potent extracts (P3-86A, K30, P3-37B, K18, 2 and P3-37C) were also evaluated for apoptosis at 24 h (Figure 1C), and chosen for further investigation to confirm the pathway of induced apoptotic cell death in HeLa cells. The cells were also evaluated for their morphological features of apoptosis using microscopy. Visual inspection showed that the morphological changes were visible within few hours after treatment of certain extracts (Figure 1C).

### Effects of extracts on MMP

**Figure 2:**
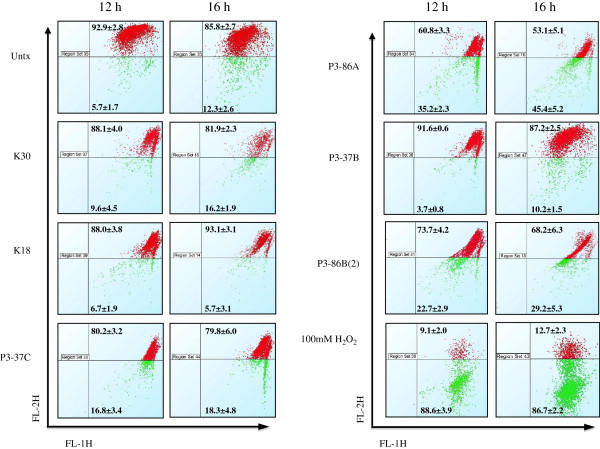
**MMP changes in HeLa cells after treatment with marine bacterial extracts.** Cells were treated with 500 μg/mL of each extract for 12 and 16 h and stained with 50 μM JC-1 and analyzed by flow cytometry. Figure presents the 2D plots of FL-2H vs. FL-1H indicating percentage of cells (± S.D) with intact (red) or permeable (green) mitochondrial membranes. Untx represent untreated sample and 100 mM H_2_O_2_ was used as a positive control. The Z- factor was calculated to be 0.79 and 0.80 for 12 h and 16 h time points, respectively.

The changes in MMP were used to evaluate its role in initiating apoptosis. In the present study, MMP was assessed using JC-1 dye. The JC-1 is a membrane permeable dye that has a unique characteristic of attraction to negative charge potential. The electron transport chain in energized mitochondria (normal MMP) attracts JC-1 dye into mitochondria where it accumulates to form J-aggregates (fluoresces red at 595 nm), while mitochondria with disrupted membrane potential (apoptotic cells) cannot accumulate JC-1, thus leaving the dye in the monomeric form (fluoresces green at 530 nm). Extracts from *Chromohalobacter salexigens* (P3-86A and P3-86B(2)) were only able to induce changes in MMP by 45% and 29% respectively (Figure 2) confirming their role in mitochondrial-mediated apoptosis.

### Activation of caspases in response to treatment with extracts

**Figure 3:**
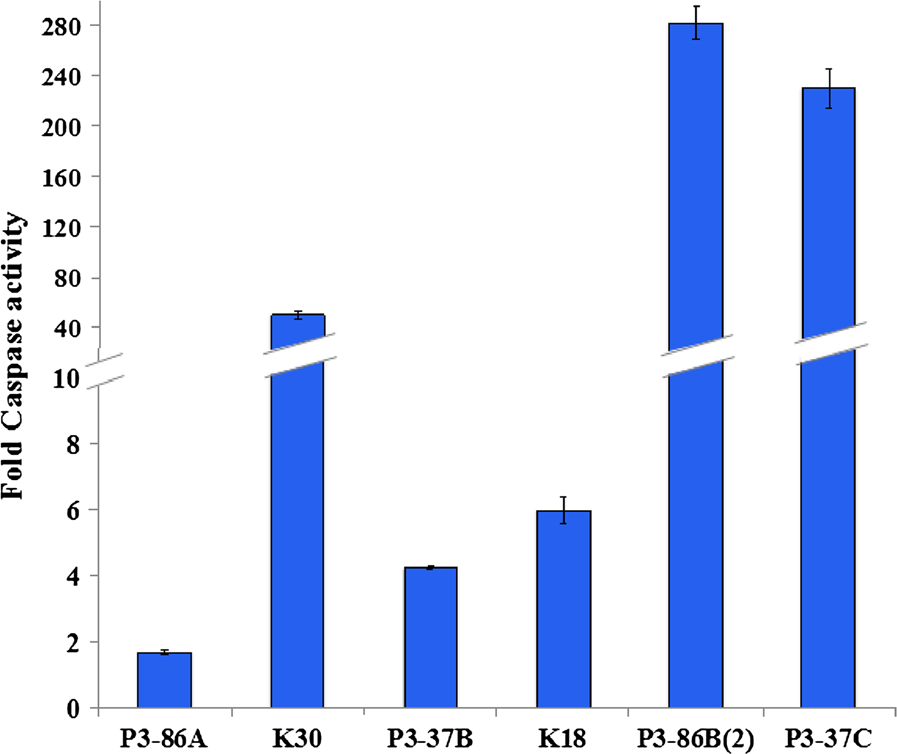
**Caspase-3/7 activity in HeLa cells after treatment with marine bacterial extracts.** Cells were treated with 500 μg/mL extracts for 16 h and caspase-3/7 activity was estimated by measuring luminescence using ApoTox-Glo kit (Promega). The caspase-3/7 activity is represented as fold-change in activity when compared to untreated cells.

To gain insights into the potential mechanisms of apoptosis involved, caspase-3/7 activity as well as protein expression of caspase-8 and −9 were measured for the six most potent extracts in HeLa cells after 16 h of treatment. All six extracts were able to activate caspase-3/7 and can be grouped further into two categories of ‘active’ and ‘highly active’ depending on the fold increase in observed caspase-3/7 activity as compared to untreated cells (Figure 3). Microbial extracts from P3-86A, P3-37B and K18 showed <10 fold increase in caspase-3/7 activity and were termed as active (in the range of 1.7-6.0 folds) while extracts from *Chromohalobacter salexigens* (K30, P3-86B(2)) and *Idiomarina loihiensis* (P3-37C) were considered highly active due to their remarkably high caspase-3/7 activity (≥50 fold increase) as compared to untreated cells. All extracts except *Chromohalobacter salexigens* (P3-86A) showed significant reduction in full-length caspase-9. Similarly, cleavage of caspase-8 (cl-Caspase-8) was observed in cancer cells treated with all other extracts except *Chromohalobacter salexigens* extract (K30).

### PARP-1 cleavage through caspases

**Figure 4:**
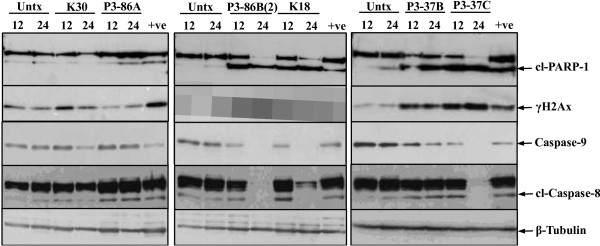
**Western blot analysis of PARP-1 cleavage, phosphorylated γH2AX, Caspase-8 and −9 in HeLa cells after treatment with marine bacterial extracts.** Protein lysates of HeLa cells treated with 500 μg/mL of each extract for 12 and 24 h were subjected to western blotting probing for cleaved PARP-1 (cl-PARP-1), procaspase-9, cleaved caspase-8 and γH2Ax. Cells treated with 400 nM docetaxel for 24 h were used as a positive (+ve) control, Untx represent untreated control and β-tubulin was used as a loading control.

The concerted action of caspases-3 and -7 lead to PARP-1 cleavage in response to DNA damaging agents [38,39] and is considered as a hallmark of apoptosis [40,41]. To further explore that induced apoptosis in HeLa cells was via PARP-1 cleavage, western blotting was performed. Figure 4 shows an elevation in the cleaved fragment of PARP-1 (85 kDa) in a time dependent manner for the extracts from *Chromohalobacter salexigens* (P3-86B(2)), *Chromohalobacter israelensis* (K18), *Halomonas meridiana* (P3-37B) and *Idiomarina loihiensis* (P3-37C). The PARP-1 cleavage is quite significant after 12 h of treatment; however only a cleaved fragment was noticeable for these extracts (except for P3-37B) at 24 h. These observations confirmed the involvement of caspases mediated PARP-1 cleavage in response to the treatment with these four marine extracts in HeLa cells.

### Activation of γH2Ax, a DNA damage marker

γH2Ax is a variant of H2A histone and is phosphorylated at serine 139 in the presence of DNA double-stand breaks caused by DNA damage [42] and DNA fragmentation during apoptosis [43]. Substantial DNA damage was measured in HeLa cancer cells within 12 h of treatment with extracts P3-37B, P3-37C, P3-86B(2) and K18 (Figure 4), confirming their role as DNA damaging agents.

## Discussion

In the present study, 24 extracts of marine bacteria isolated from the deep-sea brine pools of the Red Sea were evaluated for their cytotoxic effects against three human cancer cell lines. Out of all, 13 extracts were found to be significantly active against one or more cancer cell lines indicating their cell line specific behavior. The cell line specific activity of the extracts may be due to the presence of particular secondary metabolites and/or the different mechanisms of action of programmed cell death prevalent in different cancer cell lines.

Apoptosis or programmed cell death in multicellular organisms maintain the homeostasis by eliminating unwanted or defective cells [44]. It is well known that inefficient apoptosis contribute to several human malignancies [45,46]; therefore, the identification of anticancer agents that induce cell death via apoptosis is one of the attractive strategies for chemotherapy [47]. The extracts from *Chromohalobacter salexigens* (P3-86A, K-30, P3-86B(2)), *Halomonas meridian* (P3-37B), *Idiomarina loihiensis* (P3-37C) and *Chromohalobacter israelensis* (K-18) were found to be most actively inducing apoptosis in HeLa cells (Figure 1). These extracts induced either one or more apoptosis related molecular changes such as cell shrinkage, PS exposure by membrane flipping, caspase-3/7, -8 or -9 activation, PARP-1 cleavage (representing DNA fragmentation) and increase in phosphorylation of γH2Ax (indicating DNA damage). Not much work has been published on the isolation of cytotoxic compounds from these microbial species. Our group [15] and others [48-50] have shown previously that *Halomonas* species produce metabolites that have the potential to kill cancer cells. *Idiomarina loihiensis* is an aerobic heterotrophic bacterium capable of catabolizing amino acids as a primary energy source [23]. In PubMed, there are only 10 articles on *Idiomarina loihiensis* and most of these focus on describing its isolation and characterization [51], metabolism [23], and biofilm forming capabilities [52]. No study to date has focused on evaluating the bioactive potential of this species.

In the present study, extract from *Idiomarina loihiensis* (P3-37C) displayed caspase-dependent apoptosis in HeLa cells where a strong (229 fold) increase in caspase-3/7 activity was observed. Extract from K-18 also induced caspase-dependent apoptosis in our study, which showed 100% similarity to *Chromohalobacter israelensis. Chromohalobacter israelensis* is a euryhaline halophile shown to change its concentration of unsaturated fatty acids in response to change in salt concentration, thus providing a mechanism for halophiles to tolerate environmental stresses [53]. Nothing has been reported so far regarding cytotoxic potential of this strain. Isolates P3-86A, K-30 and P3-86B(2) were found to have high 16 s similarity with *Chromohalobacter salexigens*. This is one of the most investigated strain (out of the four we are reporting here) as a PubMed search on 15th July 2013 displayed 33 articles on *Chromohalobacter salexigens*. The Work to date has focused broadly on compatible solutes [54-57] and metabolism [58-61]. To the best of our knowledge, no attempt has been made to assess the cytotoxicity potential of these bacteria.

The key objectives of the present study were to estimate the proapoptotic potential of novel halophytes isolated from the brine pools of the Red Sea and to shed light on the mechanism of apoptosis induction in cancer cells. We investigated the mode of induction of apoptosis by marine bacterial extracts by targeting the intrinsic and extrinsic pathways in human cervical cancer cell line (HeLa). Broadly, apoptosis is known to work through two pathways, i.e., mitochondria-mediated intrinsic pathway and death receptors mediated extrinsic pathway [62]. Intrinsic pathway is activated by either permeabilization of the outer membrane of mitochondria leading to disrupted MMP, or via DNA damage. Both routes activate caspase-9 and consequently lead to activation of caspase-3 [63]. Extrinsic pathway involves interaction of ligands (Fas and TNF-alpha) to their transmembrane receptors [64], thus activating caspase-8, which further activates caspase-3 directly [65] or by first activating intrinsic pathway followed by activation of caspase-3 [66]. Intrinsic- and extrinsic-pathways merge at caspase-3, which further cleaves PARP-1 and results in apoptosis [67]. The results of pathway level investigations of the marine bacterial extracts are summarized in Table 3.

**Table 3:** Summary of the results of PS exposure apoptosis assay, Caspase-3/7 activity, MMP changes, PARP-1 and Caspase-8 cleavage, full-length Caspase-9 and γH2Ax protein expression in HeLa cells treated with 500 μg/mL concentration of extracts

**Extract**	**PS exposure (percentage of stained cells)**	**Caspase-3/7 activity (fold change)**	**MMP**	**PARP-1 cleavage**	**γH2Ax**	**Caspase-9 reduction**	**Caspase-8 cleavage**
P3-86A	+ (73.95%)	+ (1.7)	+	+	-	-	+
K30	+ (86.74%)	+ (50.0)	-	-	+	+	-
P3-37B	+ (84.89%)	+ (4.2)	-	+	+	+	+
K18	+ (85.85%)	+ (5.9)	-	+	+	+	+
P3-86B(2)	+ (80.01%)	+ (282.1)	+	+	+	+	+
P3-37C	+ (84.77%)	+ (229.7)	-	+	+	+	+

‘+’ represents a positive observation, whereas ‘-’represents a negative observation.

We reveal here that extracts from *Chromohalobacter salexigens* (P3-86A and P3-86B(2)) induced MMP disruption, caspase-3/7 activation, PARP-1 cleavage and PS exposure. PS externalization represents an early event during execution phase of apoptosis occurring between caspases activity and nuclear condensation [68]. Further investigation into the expression of caspase-8 and −9 determined the cleavage of caspase-8 after treatment with extract P3-86A, while no change in expression of full-length caspase-9 was observed, (Figure 4). This confirms that P3-86A induces apoptosis through extrinsic pathway. Extract P3-86B(2) was found to reduce expression of both full-length caspase-8 and −9 (Figure 4), thus suggesting that both extrinsic and extrinsic pathways of apoptosis are involved in its mechanism of action. The extracts from *Halomonas meridiana* (P3-37B), *Chromohalobacter israelensis* (K18) and *Idiomarina loihiensis* (P3-37C) were unable to induce any change in MMP in HeLa cancer cells and thus suggest the mitochondrial-independent apoptotic induction. The expression of both full-length caspase-8 and −9 was significantly reduced thus confirming the involvement of these initiator caspases in apoptosis induction. DNA damage was also observed in cancer cells which is known to activate Caspase-9 [69,70] leading to intrinsic apoptosis in the absence of mitochondrial-mediated pathway. In case of *Chromohalobacter salexigens* (K30) extract, in spite of a 50-fold increase in caspase-3/7 activity and PS externalization, K-30 neither caused any change in MMP nor cleavage of PARP-1, although a slight increase in γH2Ax was observed indicating DNA damage. It is well documented that PARP activity is induced in response to DNA strand breaks in cells that have been exposed to DNA-damaging agents [71]. Although it is widely accepted that PARP is specifically cleaved during apoptosis [72] by caspase-3 [73] and caspase-7 [74], but studies have also shown that PARP activity [75], activation of PARP-cleaving enzymes [76] and cleavage of PARP-1 [77] are not essential for induction of apoptosis. In another study, uncleavable PARP has been shown to accelerate apoptosis and necrosis [78] with possible explanation that uncleavable PARP may lead to imbalanced energy pool by depleting NAD + and ATP pools, which further disrupts MMP, thus releasing proapototic factors from mitochondria. In our study, K30 did not disrupt MMP and hence the above mentioned explanation does not clarify the mechanism of apoptosis induction by K30. Caspase-9 was significantly reduced at 24 h after K30 induction. This suggests that the K30 induces apoptosis in cancer cells through intrinsic pathway where DNA damage leads to activation of caspase-9 that further contributes to the observed activities of caspase-3/7 and PS exposure.

In the last decade, phosphorylated gamma-H2AX (γH2Ax) has emerged as a marker of DNA damage [79] and drug response in cancer patients [80,81]. The chemicals/drugs that lead to DNA damage in cells are known as ‘genotoxic drugs’. Several genotoxic compounds such as cisplatin, carboplatin, oxaliplatin, methotrexate, doxorubicin, daunorubicin etc., are currently being used in the treatment of various types of cancers [82]. The extracts tested in the present study also showed strong DNA damage as measured using γH2Ax, which shows that these extracts may contain compounds that could find potential therapeutic use in cancer patients. This study opens up avenues for identifying new DNA damaging compounds from deep-sea bacteria.

## Conclusions

This study reports for the first time the cytotoxic activities of several halophilic bacterial species isolated from deep sea brine pools of the Red Sea and provides in-depth insights into the possible mechanisms of apoptosis induced by the extracts in various human cancer cell lines. Overall, six extracts from *Chromohalobacter salexigens* (P3-86A, K-30, P3-86B(2)), *Halomonas meridian* (P3-37B), *Idiomarina loihiensis* (P3-37C), and *Chromohalobacter israelensis* (K-18) have displayed significant anticancer activities and can be further explored for isolation and characterization of bioactive molecules. This study also provides conclusive evidence that brine pools of the Red sea harbor several species of bacteria producing anticancer secondary metabolites.

## Abbreviations

CTD: Conductivity-temperature-depths; TFF: Tangential flow filtration; DMEM: Dulbecco’s modified eagle’s medium; FCS: Fetal calf serum; OD: Optical density; MTT: 3-(4, 5-Dimethylthiazol-2-yl)-2, 5-diphenyltetrazolium bromide; PS: Phosphatidylserine; HeLa: Cervical carcinoma; MCF-7: Breast adenocarcinoma; DU145: Prostate carcinoma; MMP: Mitochondrial membrane potential; PARP: Poly(ADP-ribose) polymerase.

## Competing interest

The authors declare that they have no competing interests.

## Authors’ contribution

SS and MK planned the study, wrote manuscript and performed experiments along with LE. TH and GH isolated the strains and provided taxonomic classification of the bacterial strains. KH was responsible for growth of the strains in large batches. US was responsible for the planning of the expedition and the cultivation experiments, provided general coordination of the study and helped in manuscript writing. VBB provided general coordination of the study. All authors read and approved the final manuscript.

## Pre-publication history

The pre-publication history for this paper can be accessed here:

http://www.biomedcentral.com/1472-6882/13/344/prepub
